# Case report: MR spectroscopy in pantothenate kinase-2 associated neurodegeneration

**DOI:** 10.4103/0971-3026.69353

**Published:** 2010-08

**Authors:** Umesh C Parashari, Pallavi Aga, Anit Parihar, Ragini Singh, Vindhya Joshi

**Affiliations:** Department of Radio Diagnosis, C.S.M. Medical University, (Upgraded K.G. Medical University) Lucknow, UP, India

**Keywords:** Basal ganglia, dystonia, Hallervorden–Spatz disease, iron deposition, magnetic resonance spectroscopy, PKAN

## Abstract

We report a case of a 13-year-old girl with Hallervorden-Spatz disease (HSD) or pantothenate kinase-2 associated neurodegeneration (PKAN). HSD is a rare neurodegenerative disorder, which is characterized by a rapidly progressive extrapyramidal syndrome, dementia with optic atrophy, and retinal degeneration. It is associated with accumulation of cysteine-iron complex in the globus pallidi and substantia nigra. The MRI “eye of the tiger” sign is the characteristic. MRI spectroscopy is also characteristic. It shows markedly decreased NAA/Cr values in the globus pallidi and substantia nigra with increased mI/Cr values that suggest of gliosis.

## Introduction

Pantothenate kinase-2 associated neurodegeneration (PKAN) or Hallervorden-Spatz disease (HSD) is a rare autosomal recessive degenerative disorder. In PKAN, neurons of the globus pallidus and substantia nigra are affected due to excessive iron deposition.[1–3] The disease is characterized by childhood onset of progressive dystonia, rigidity, choreathetosis, dysarthria, mental changes, and visual disturbances. Recently, a defect in a pantothenate kinase gene (*PANK2*) has been demonstrated in these patients; the gene is localized to chromosome 20p12.3-13 and codes for pantothenate kinase-2.[1,2] The “eye-of-the-tiger” appearance is the characteristic finding described on MRI.[1,2,4] Iron deposition causes gliosis in the affected area.[5] There has been a limited description of the role of MRI spectroscopy (MRS) in PKAN.[6–8] We present here a case where MRS helped in the identification and quantification of axonal involvement in a case of PKAN.

## Case Report

A 13-year-old girl came with complaints of progressively increasing abnormal movements, intellectual decline, and slowing of voluntary movement. She was not able to continue her studies because of her complaints. She was the product of a consanguineous marriage and had been born at full term. There was history of normal development till the age of 8 years. A history of perinatal hypoxic insult or birth trauma was absent. There was no other relevant family history.

Neurological examination revealed a generalized increase in the tone in all four limbs, with dystonic arching of the trunk; mild hyperreflexia, with extensor plantar responses; choreoathetotic movements in the upper extremities; and tremors in the tongue, with speech deterioration. On ocular examination, nystagmus was present on lateral gaze. Ophthalmic evaluation was normal. No evidence of a Kayser-Fleischer ring was seen on slit-lamp examination. Serum electrolytes, iron, copper, and ceruloplasmin levels were within normal limits. Amino acid chromatographic analysis was normal. A few acanthocytes were seen in the blood smear.

**Figure 1 (A,B):**
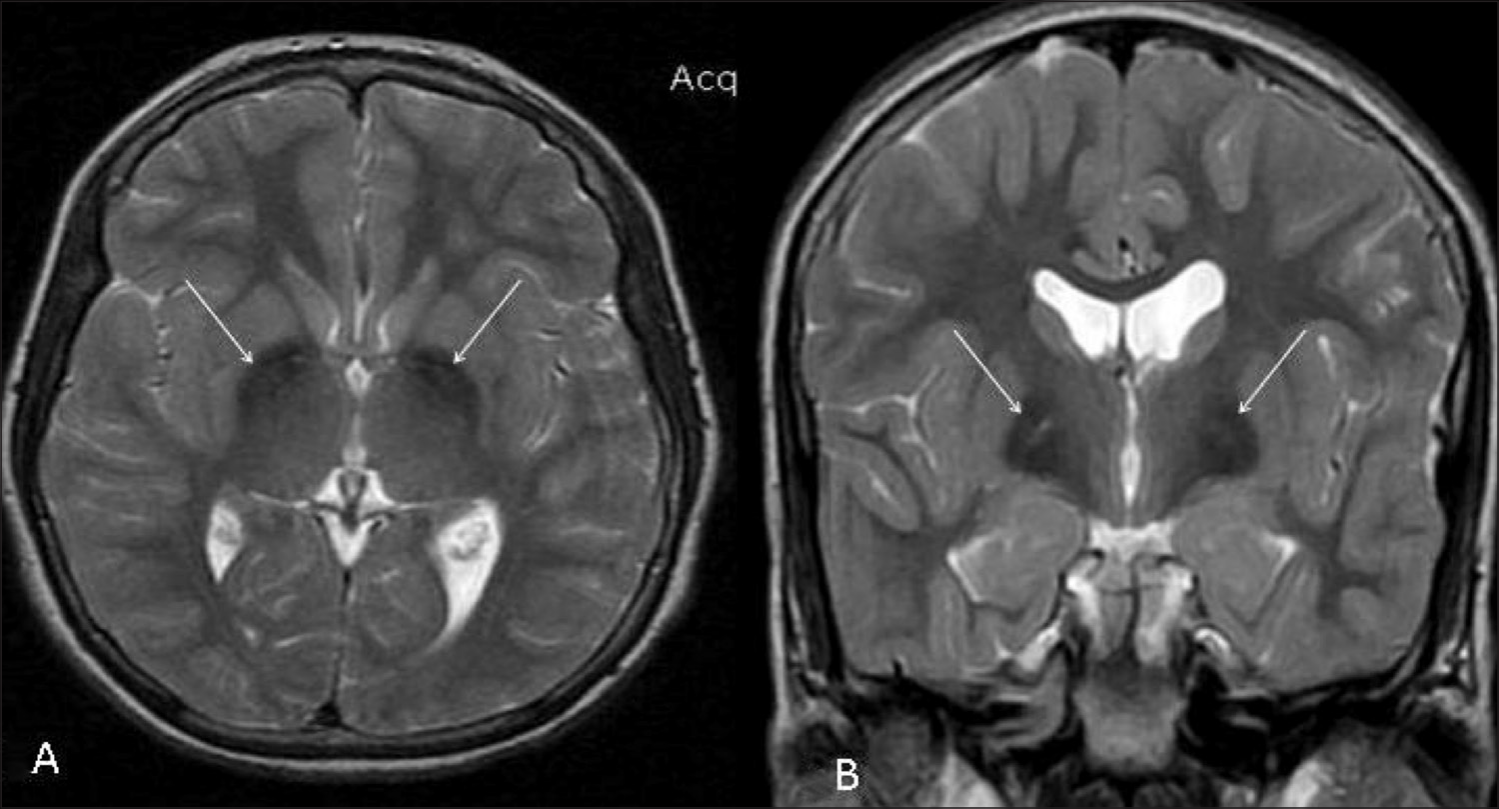
T2W axial (A) and coronal (B) MRI images show the classic “eye-of-the-tiger” sign, with marked hypointensity within both globus pallidi with a small area of central hyperintensity (arrows). Note that the sign is better appreciated on the coronal image

**Figure 2:**
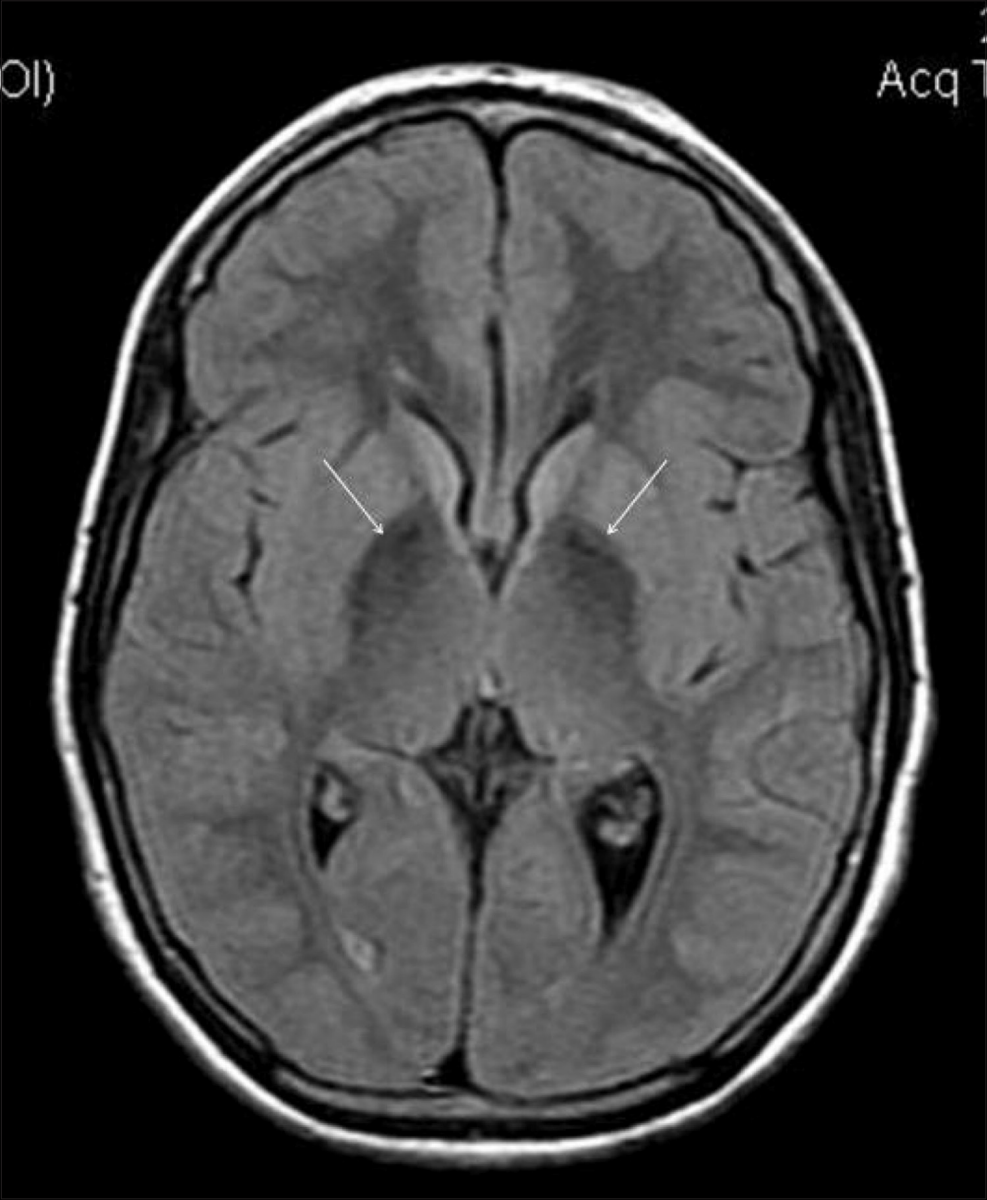
Axial FLAIR MRI image shows hypointense signals (arrows) within both globus pallidi

**Figure 3 (A,B):**
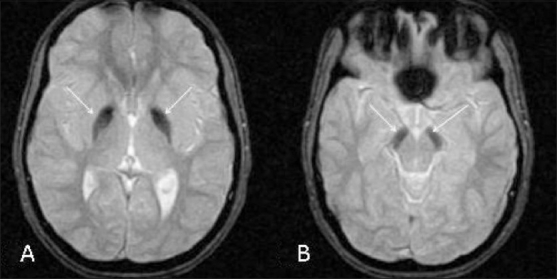
Axial T2W gradient-recalled echo MRI images show marked hypointensity in the globus pallidi (arrows in A) and parsreticulata of the substantia nigra (arrows in B)

MRI examination was performed on a 1.5T scanner (GE Medical Systems, Milwaukee, USA). MRI showed marked hypointensity within both globus pallidi, with a small area of central hyperintensity (eye-of-the-tiger sign) on T2W images [Figure 1AB]. Similar hypointense signals were also seen on the FLAIR images [Figure 2]. The marked hypointensity in the globus pallidi was better appreciated on T2W gradient-recalled-echo (GRE) images due to the susceptibility effect, suggesting iron deposition [Figure 3A]. Similar T2-hypointense lesions were seen in the substantia nigra [Figure 3B]. Mild thinning of the corpus callosum was noted in the region of the body. Based on the clinical assessment and the typical MRI findings, we arrived at the diagnosis of PKAN.

**Figure 4 (A-C):**
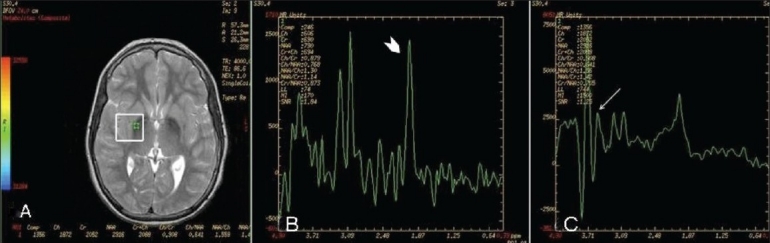
MRI spectroscopy performed with an ROI (region of interest) circle placed in the right globus pallidus (A). The spectra reveal a markedly decreased NAA peak (white arrowhead in B), with a decreased NAA/Cr and an increased myoinositol peak (thin white arrow in C) in the globus pallidi

To evaluate the neuroaxonal degeneration quantitatively, proton MRS was performed using multivoxel chemical-shift imaging with spin-echo, point resolved spectroscopy (PRESS). The imaging parameters were as follows: TR - 4000 ms; TE - 86.6 and 35 ms; slab thickness - 10 mm; voxel size - 20 × 20 × 20; number of acquisitions - 2, and scan time - 6.0 min. Automated software was used for the calibration of the spectrum. Spectra were taken with the ROI (region of interest) circle placed in the right globus pallidus [Figure 4A], which showed the following values; *N*-acetylaspartate(NAA)/creatine(Cr): 1.14 and myoinositol(mI)/Cr: 0.73. The study revealed a decreased NAA peak [Figure 3B] and a reduced NAA/Cr ratio [Figure 4B], suggesting neuroaxonal loss. An increased myoinositol peak (thin white arrow) and mI/Cr ratio were seen on MRS done at a TE of 35 ms, suggestive of glial proliferation [Figure 4C].

## Discussion

PKAN is a rare neurodegenerative disorder that was first described by Hallervorden and Spatz in 1922.[9] The inheritance pattern is autosomal recessive. On an average, the diagnosis is usually made in the 1st decade of life or in early adolescence.[10] After diagnosis, average survival is for about 12 years.[11]

The globus pallidus, subthalamic nuclei, and pars reticulata of the substantia nigra are normally rich in iron. Aberrant storage of iron is an essential factor in the causation of PKAN.[1–3] Excess deposition of iron causes neuronal degeneration, gliosis, and spheroid formation (vacuolization).[4] The characteristic MRI findings of bilateral symmetrical hyperintense signals surrounded by hypointensity on T2W images lead to the “eye-of-the-tiger” sign.[1,2,4] The surrounding hypointensity is caused by signal loss (susceptibility) from the iron deposition, while the central hyperintensity is due to axonal swelling, formation of spheroids, gliosis, and neuronal loss and degeneration.[5] Although this finding is considered specific for PKAN, it can be found in other parkinsonian syndromes as well. The MRI findings correspond well with the histopathological changes.[5] Gliosis and spongiosis appear hyperintense on T2W images, while iron deposition appears hypointense due to susceptibility-related signal loss and is better appreciated on GRE images.

The locus of the causative gene is 20p12.3-13 and it codes for pantothenate kinase-2 (PANK2).[1,2] PANK2 is required for the phosphorylation of pantothenic acid in the formation of coenzyme A. Defective phosphorylation causes underutilization of cystine which, when present in excess, chelates iron, resulting in free radical formation. Excessive presence of pantothenate kinase receptors is responsible for the preferential involvement of the globus pallidi, subthalamic nuclei, and pars reticularis of the substantia nigra.

Recently, Hayflick *et al*.[1] on the basis of the age of onset and the gene defect present have classified neurodegenerative disorders of the brain with iron accumulation into different groups. The classical form, with the PANK2 mutation, is characterized by early onset, rapid progression, and the presence of the typical eye-of-the-tiger sign. Atypical disease is characterized by late onset and slow progression. In patients with atypical disease, PANK2 mutation is present in only 33% of cases.[4] The eye-of-the-tiger sign may or may not be present in these patients.

For the quantitation of neuroaxonal degeneration, proton MRS has been used.[6–8] Metabolites evaluated are N acetyl aspartate (NAA) and myoinositol (mI). NAA is a neuronal marker and is almost exclusively present in neuronal tissues, while mI is a marker of glial proliferation.[6–8] The NAA peak is evaluated at both low and high TE while the mI peak is evaluated at low TE (30 ms). The NAA/Cr ratio is used for the quantitation of neuroaxonal degeneration in affected regions of the brain. In neurodegenerative disorders, a reduction in the NAA/Cr ratio occurs.[6–8] An Increased mI/Cr ratio has also been described in patients of PKAN.[6–8] An increase in mI is suggestive of gliosis.

Our patient was classified as classical PKAN disease on the basis of the age of onset, clinical evaluation, and the specific pattern demonstrated on MRI and MRS.

The differential diagnosis in cases of iron deposition in the basal ganglia and the “eye-of-the-tiger” sign, includes aceruloplasminemia and neuroferritinopathy. These conditions present in adults. Association of diabetes mellitus has been reported in aceruloplasminemia, along with deficiency of the ceruloplasmin protein. The locus for ceruloplasmin protein is chromosome 3q13.3. The age of onset in neuroferritinopathy is approximately the 5^th^to 6^th^decade. A few other metabolic disorders, such as organic aciduria, cortical basal ganglionic degeneration, and early-onset levodopa-responsive parkinsonism, also show hyperintense signals within the basal ganglia. Wilson disease, Leigh disease, infantile bilateral necrosis, and mitochondrial encephalopathies also show involvement of the lentiform nucleus, but in these disorders the putamen is predominantly involved rather than the globus pallidus.[12]

At present, there is no specific treatment for PKAN and the management is symptomatic.[1,2] Recently, iron chelators such as VK-28 have been use for decreasing basal as well as iron/ascorbate-induced mitochondrial lipid peroxidation in rats[13] Serial MRS can be used for the follow-up of PKAN patients on iron chelators to quantitatively assess the axonal damage and gliosis. The medical treatment of PKAN is ineffective, prolonged, and has many side effects and so surgical modalities like stereopallidotomy and thalamotomy[14] have also been used to reduce dystonia. The benefits however are short lasting with many side effects and complications. Future management strategies may involve direct delivery of phosphorylated pantothenate to the cells, bypassing pantothenate kinase[15]
